# Applications, Shortcomings, and New Advances of Job Safety Analysis (JSA): Findings from a Systematic Review

**DOI:** 10.1016/j.shaw.2023.03.006

**Published:** 2023-03-11

**Authors:** Fakhradin Ghasemi, Amin Doosti-Irani, Hamed Aghaei

**Affiliations:** 1 Occupational Health and Safety Engineering Department, Abadan University of Medical Sciences, Abadan, Iran; 2 Department of Epidemiology, School of Public Health and Research Center for Health Sciences, Hamadan University of Medical Sciences, Hamadan, Iran; 3 Department of Occupational Health Engineering, School of Health, Arak University of Medical Sciences, Arak, Iran

**Keywords:** Accident prevention, Occupational safety, Safety analysis

## Abstract

**Background:**

Job safety analysis (JSA) is a popular technique for hazard identification and risk assessment in workplaces that has been applied across a wide range of industries. This systematic review was conducted to answer four main questions regarding JSA: (1) which sectors and areas have used JSA? (2) What has been the aim of employing JSA? (3) What are the shortcomings of JSA? (4) What are the new advances in the field of JSA?

**Methods:**

Three main international databases were searched: SCOPUS, Web of Science, and PubMed. After screening and eligibility assessment, 49 articles were included.

**Results:**

Construction industries have used JSA the most, followed by process industries and healthcare settings. Hazard identification is the main aim of JSA, but it has been used for other purposes as well. Being time-consuming, the lack of an initial list of hazards, the lack of a universal risk assessment method, ignoring hazards from the surrounding activities, ambiguities regarding the team implementing JSA, and ignorance of the hierarchy of controls were the main shortcomings of JSA based on previous studies.

**Conclusion:**

In recent years, there have been interesting advances in JSA making attempts to solve shortcomings of the technique. A seven-step JSA was recommended to cover most shortcomings reported by studies.

## 1 Introduction

**Fig. 1:**
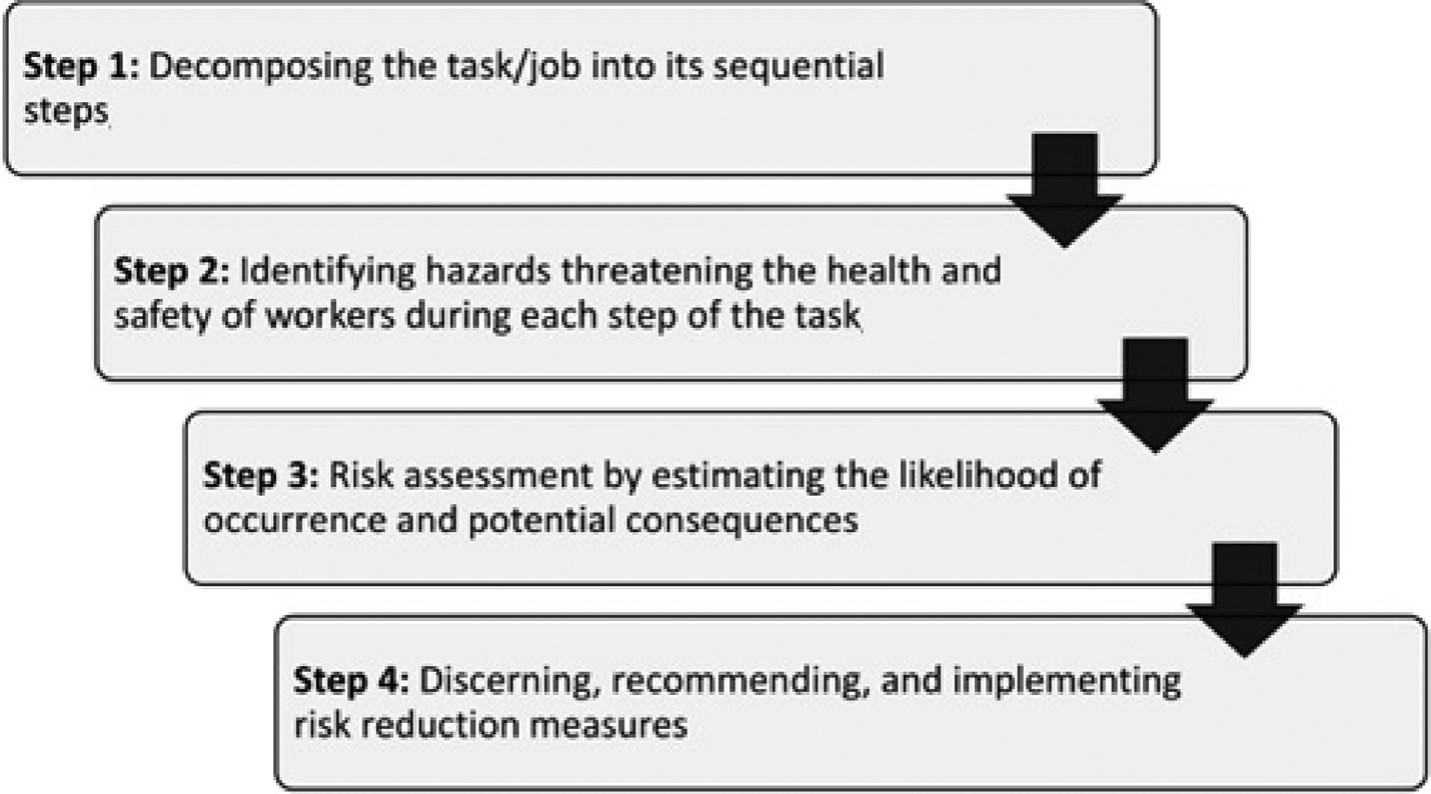
General steps of JSA. JSA, job safety analysis.

Job safety analysis (JSA) is hazard identification and sometimes qualitative risk assessment method, which specifically concentrates on hazards created by the task performed by an employee [1]. Job hazard analysis, job safety assessment, job hazard assessment, task safety assessment, task hazard assessment, task safety analysis, and task hazard assessment all are other terms used interchangeably by literature. Regardless of the term used, the technique is aimed at identifying the existing and potential hazards of a task/job, assessing their risks, and preventing losses by recommending and implementing effective control measures [2]. JSA is supposed to be rooted in the job analysis offered by Taylor's scientific management approach [3,4]. Heinrich [3,4] for the first time employed the term JSA to highlight the safety aspects of job analysis. The technique is normally conducted in four main steps as shown in Fig. 1.

In addition to safety and health purposes, JSA can be utilized for improving product quality by identifying and eliminating potential human errors and determining skills needed to perform a task [2]. JSA is an integral part of any safety management system. If performed appropriately, it can provide employees and employers with an in-depth understanding of how work should be done to prevent adverse outcomes, based on which safety procedures and instructions can be developed. Determining safety training contents is another purpose of JSA [5]. Many retrospective studies have found that implementing a proper JSA is effective in preventing accidents [6,7] and promoting safety behavior [8]. Albrechtsen et al [9] asserted that JSA has at least six benefits: (1) formalization of work, (2) accountability, (3) participation of employees, (4) organizational learning, (5) hazard identification and situation awareness, and (6) loss prevention.

Over the years, JSA has become popular in various industries and service companies. However, there are some questions related to this method, which can be well answered only by conducting a systematic review. First, which industries and industrial operations have used JSA the most? For example, it is evident that JSA is applicable for most operations at construction sites, but it is not clear which operations in a process industry can benefit from JSA. Second, there is an inconsistency across studies about the purpose of implementing this technique. JSA is a technique originally developed for hazard identification, but it also has been used for other purposes. Consequently, the second purpose of this study was to clarify the objectives that can be accomplished by operationalizing JSA. Third, although JSA is a technique originally for hazard identification, it has been extensively used for risk assessment too. As there is no universally accepted risk assessment method for the hazards identified by JSA, this systematic review was conducted to investigate and summarize risk assessment approaches employed in conjunction with JSA. Forth, similar to other hazard identification and risk assessment techniques, JSA may suffer from an array of shortcomings that are presently reported by other studies in a sporadic manner. Summarizing these downsides can provide both researchers and practitioners with a quick guide to use the technique correctly and cautiously. Fifth, there have been a number of advances in how JSA is designed, implemented, and its results are used. This study also aimed at making an overview on the latest advancements regarding this technique.

## 2 Material and methods

For conducting a systematic review, a standard and well-accepted approach should be adopted in order to avoid biases [10]. This systematic review was conducted based on the Preferred Reporting Items for Systematic reviews and Meta-Analyses (PRISMA) statements [11]. Worth mentioning, the study protocol and method is reviewed and approved by ethic committee of Abadan University of Medical Sciences (ethic code: IR.ABADANUMS.REC.1401.026).

### 2.1 Search strategy

In this study, three major international databases, SCOPUS, Web of Science, and PubMed, were searched. All studies published before May 2022 were included regardless of their languages and geographical regions. Seven different terms may be used interchangeably with JSA: job hazard analysis, job safety assessment, job hazard assessment, task safety assessment, task hazard analysis, task safety analysis, and task hazard assessment. All these terms were searched and papers that used one of them in their title, abstract, or body were included. The strings searched were: “job hazard analysis” OR “job safety assessment” OR “job hazard assessment” OR “task safety assessment” OR “task hazard analysis” OR “task safety analysis” OR “task hazard assessment”. Search in the SCOPUS was based on the “title,” “abstract,” and “keywords,” in the PubMed based on the “all text,” and in the Web of Science based on the “topic.”

After removing duplications, the title and abstract of remaining records were screened by two independent researchers (F G and H A). Only peered-reviewed original articles were included. Conference papers, news, bulletins, books, book chapters, and review articles were excluded in this step. For comparing the results obtained by two researchers, the agreement percentage and the Kappa agreement coefficient were calculated.

### 2.2 Eligibility criteria

In this step, the remaining articles were scrutinized for their eligibility. The first eligibility criterion considered in this study was relevancy. Only those articles in which JSA was implemented were included regardless of their language, publication time, and the country in which the study has been conducted. Moreover, review articles, bulletins, conference papers, and all records other than original articles which were not discerned in previous steps were identified and removed. Similar to the previous step, the agreement percentage and the Kappa agreement coefficient were calculated in this step to determine the degree of agreement between the two researchers.

### 2.3 Quality assessment

Based on the study objectives and questions and the fact that the study did not aim to provide a pooled estimate, this study is of the scoping systematic review type, which is slightly different from the classic systematic reviews. The quality assessment is not obligatory for such studies; consequently, this step was not performed in this step [12].

### 2.4 Data extraction

In this step, required data were extracted based on the objectives and questions of the study. The information included the type of industry and operation for which JSA is conducted, the purpose of the JSA, the risk assessment method employed, the mentioned shortcomings, and advancements made in the traditional JSA.

## 3 Results

A total number of 348 records (SCOPUS = 272, PubMed = 19, and Web of Science = 57) were initially identified by both researchers. Sixty-one records were removed because of duplication. After initial screening, 137 conference papers, 17 books/book chapters, 8 review articles, 40 news/news articles, or bulletins, and 15 records not related to JSA were removed. Researchers agreed on 93.4% of cases and the Kappa coefficient was equal to 0.80, demonstrating a substantial agreement between the two researchers. Researchers did not agree on the inclusion of 20 records, so they were included to be further investigated. Therefore, 70 records were qualified for the next step.

After the eligibility assessment, 46 articles were included, and 17 were excluded by both researchers. The opinions of experts did not match in six cases. After discussing these records, three of them were included. One article was also excluded because of the unavailability of the full text. Accordingly, 49 articles were finally included in this study. Researchers agreed on 91.67% of cases, and the Kappa coefficient in this step was 0.79, demonstrating a substantial agreement.

**Fig. 2:**
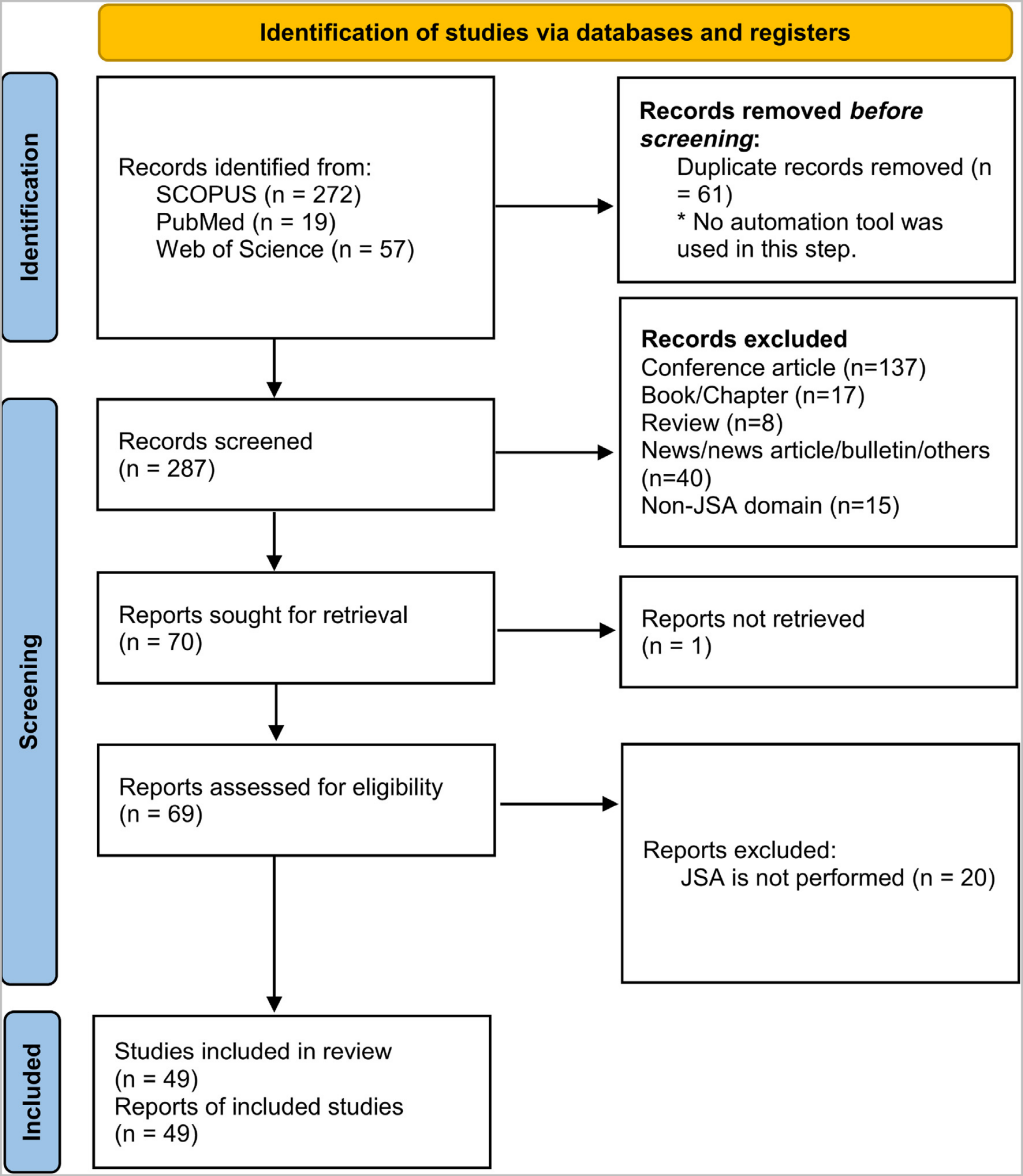
PRISMA flowchart of the systematic search. PRISMA, preferred reporting items for systematic reviews and meta-analyses.

**Fig. 3:**
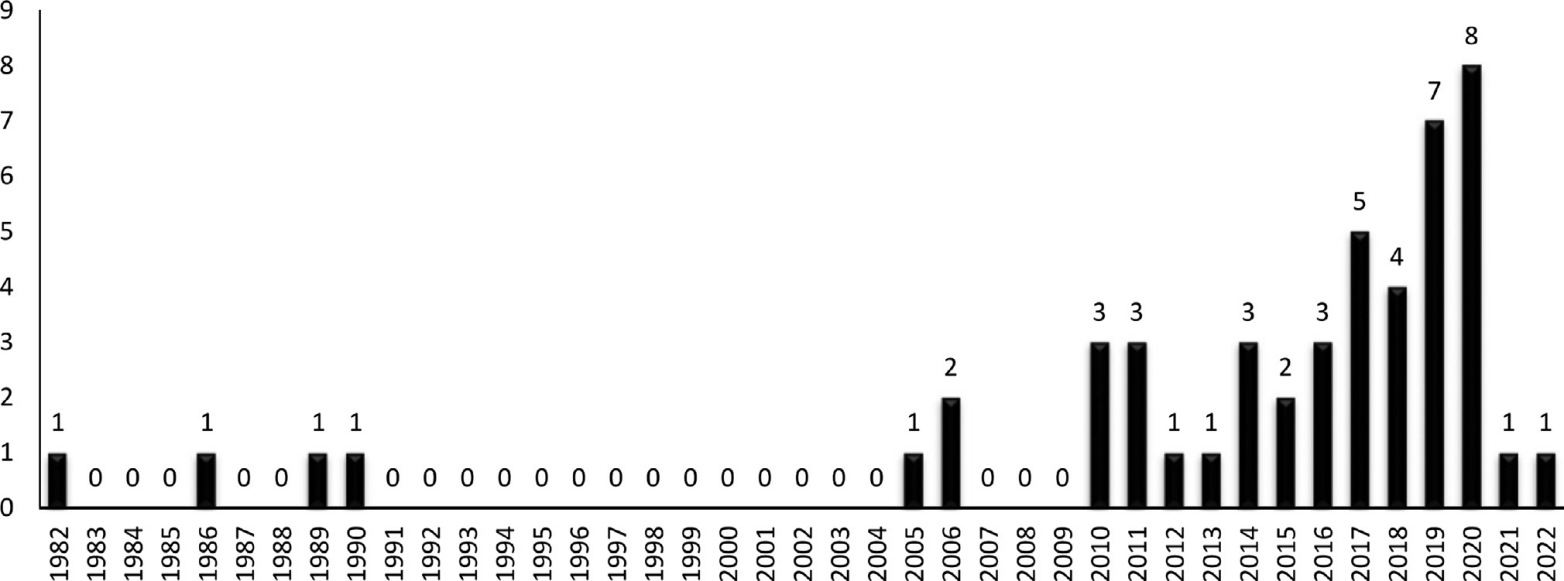
Distribution of included articles over years.

A summary of included articles is presented in Table 1. The PRISMA diagram of this review is presented in Fig. 2. Detailed information regarding the initial search, screening, and finally included papers are demonstrated in this diagram. Fig. 3 demonstrates the number of included articles each year. As evident, the number of studies has gradually increased between 1982 and 2020.

**Table 1:** A summary of included articles in the present systematic review

Source	Industry	Operation/task	Risk assessment	Objective
He et al (2022) [13]	Process industries	Hot works on gas pipelines	Self-developed	Assigning control measures
X. Li et al (2013) [14]	Process industries	Maintenance of subsea pipelines	Self-developed	Assigning control measures
W. Li et al (2019) [15]	Process industries	Gas transmission startup process	Self-developed	Improving resilience
Ghaleh et al (2019) [16]	Process industries	Road transportation of HAZMAT	Risk priority number	Assigning control measures
Li et al (2018) [17]	Process industries	Non-routine operations	Self-developed	Assigning control measures
Adekitan (2018) [18]	Process industries	Jet fuel tank corrosion recertification operation	—	Assigning control measures
Li et al (2016) [19]	Process industries	Non-routine tasks in gas transmission station	Self-developed	Developing safety procedures
Bhattacharjee et al (2014) [20]	Process industries	Phenol–formaldehyde runaway reaction	—	Incident investigation
Collins (2010) [21]	Process industries	The solvent feed line operation	—	Assigning control measures
Veley et al (2006) [22]	Process industries	Drilling rigs operations	—	Assigning control measures
Kjellen (1990) [23]	Process industries	Offshore operations	—	Assigning control measures
Yoon et al (2011) [24]	Process industries	Permit-to-work system	Self-developed	Managing permit-to-work system
Rodrigues et al (2021) [25]	Construction	Working at height	—	Assigning control measures
Pedro et al (2020) [26]	Construction	General tasks	Self-developed	Assigning control measures
Albrechtsen et al (2019) [9]	Construction	General tasks	—	Assigning control measures
Akboğa and Baradan (2017) [27]	Construction	Ready mixed concrete production	—	Assigning control measures
Zou et al (2017) [28]	Construction	Road construction	—	Assigning control measures
Zheng et al (2017) [29]	Construction	Offshore infrastructure construction	—	Assigning control measures
Zhang et al (2015) [30]	Construction	Masonry operations	—	Assigning control measures
Chi et al (2014) [31]	Construction	General tasks	—	Assigning control measures
Omar et al (2013) [32]	Construction	Preventive maintenance on green buildings	MIL-STD-E882	Assigning control measures
Ikuma et al (2011) [33]	Construction	Homebuilding	—	Improving overall performance
Wang et al (2011) [34]	Construction	General tasks	—	Assigning control measures
Patrucco et al (2010) [35]	Construction	Operating pneumatic transportation system	—	Assigning control measures
Rozenfeld et al (2010) [36]	Construction	General tasks	Self-developed	Assigning control measures
Mattila (1989) [37]	Construction	General tasks at building construction sites	—	Assigning control measures
James et al (2014) [38]	Construction	base-framing, sheet rock hanging, and painting	Self-developed	Assessing the effectiveness of a Kaizen-based intervention
Jones et al (2020) [39]	Healthcare	Intubation	—	Selection of appropriate PPE
Honarbakhsh and Jahangiri (2018) [40]	Healthcare	General tasks	Self-developed	Developing respiratory protection program
Ramsay et al (2006) [41]	Healthcare	Emergency department	—	Assigning control measures
Simon et al (2016) [42]	Healthcare	Operating room tasks	—	Assigning control measures
Palega (2021) [43]	Cutting industries	Working with laser cutter machine	Self-developed	Assigning control measures
Badoozadeh et al (2021) [44]	Printing industries	General tasks	MIL-STD-E882	Assigning control measures
Rahman et al (2020) [45]	Textile	Carpet manufacturing	Self-developed	Assigning control measures
LeChevallier et al (2020) [46]	Water treatment	General tasks	—	Selection of appropriate PPE
Levinrarian et al (2020) [47]	Transportation	Container loading and unloading	US/NZ 4360	Assigning control measures
Aditya et al (2020) [48]	Ship manufacturing	Welding	Self-developed	Assigning control measures
Suherdin et al (2020) [49]	Pharmaceutical industries	General tasks	MIL-STD-E882	Assigning control measures
Babaei Pouya et al (2019) [50]	Waste management	Informal waste pickers	MIL-STD-E882	Assigning control measures
Sholihah et al (2019) [51]	Playing facilities	Children activities	US/NZS 4360	Assigning control measures
Khoshk Daman (2018) [52]	Cable manufacturing	Main nine jobs	MIL-STD-E882	Assessing the effectiveness of a warning signs-based intervention
Halvani et al (2017) [53]	Steel manufacturing	General tasks	William Fine method	Assigning control measures
Rasoulzadeh et al (2016) [54]	Car industries	Car repair (mechanical works)	MIL-STD-E882	Assigning control measures
Park (2016) [55]	Dry-cleaning	Pressing operations	—	Assigning control measures
Bentley et al (2005) [56]	Forestry	Felling task	—	Assigning control measures
Veltrie (1987) [57]	Photoelectric industry	Emergency tasks	—	Training and emergency planning
Saarela (1982) [58]	Barrel manufacturing	Production line tasks	—	Assigning control measures
Thepaksorn et al (2017) [59]	Wood industry	Sawmill tasks	Self-developed	Assigning control measures
Dharmawirawan and Modjo (2015) [60]	Fishing	The compressor diving task	—	Assigning control measures

### 3.1 JSA applications

**Fig. 4:**
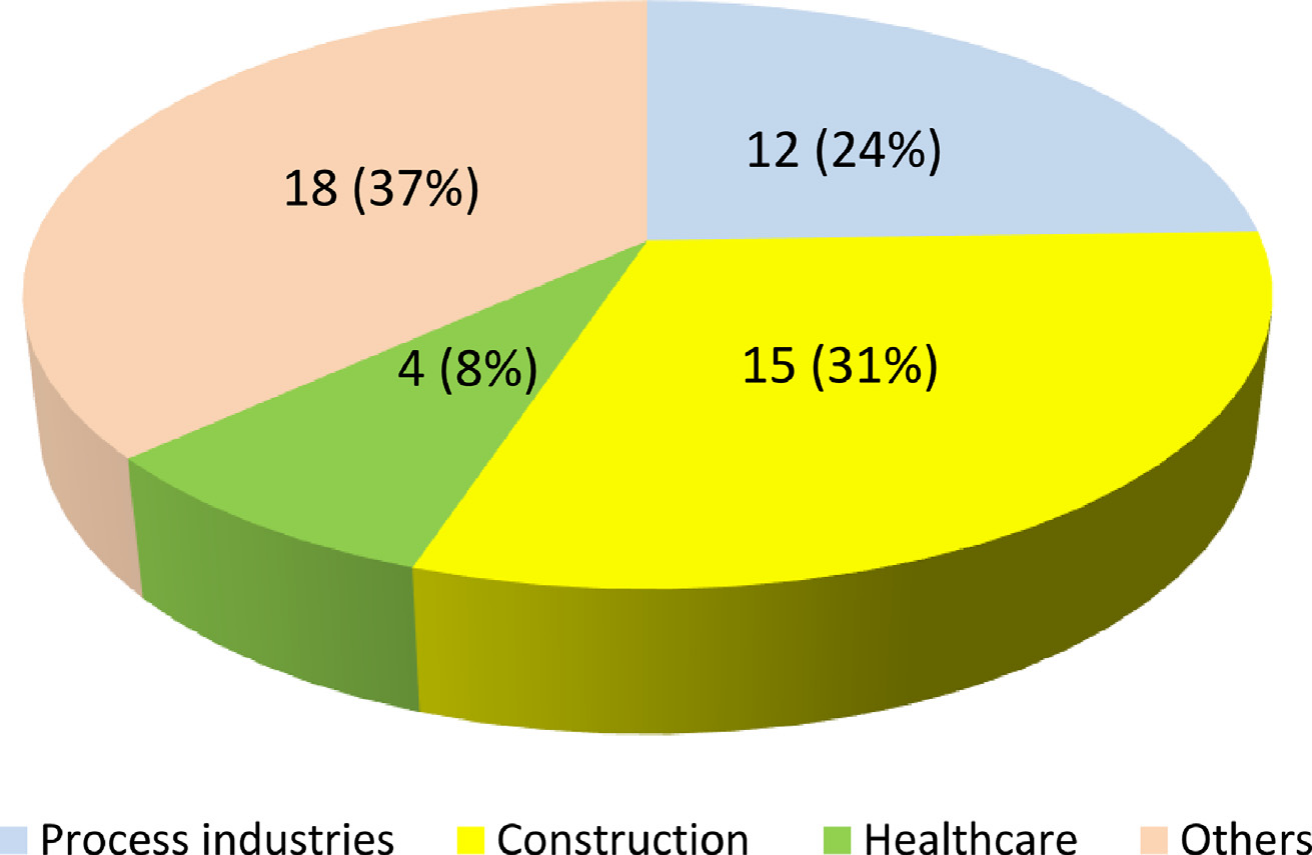
Distribution of performed JSA across various sectors. JSA, job safety analysis.

Results demonstrated that JSA has been implemented in a wide range of industries. According to Fig. 4, JSA has been mostly used in construction sites (31% cases), process industries (24% cases), and healthcare (8% cases). In construction sites, JSA has been used for most tasks and operations. In process industries, JSA acts as a supplement to process hazard assessment techniques. It has been extensively used in operations that employees play the central role, particularly in maintenance and repair works. In healthcare settings, JSA has been widely utilized for identifying hazards and risks in emergency departments, operating rooms, and patient critical cares. Moreover, JSA has been occasionally used in other sectors such as manufacturing, forestry, fishing, and so on (details are presented in Table 1).

### 3.2 JSA objectives

**Fig. 5:**
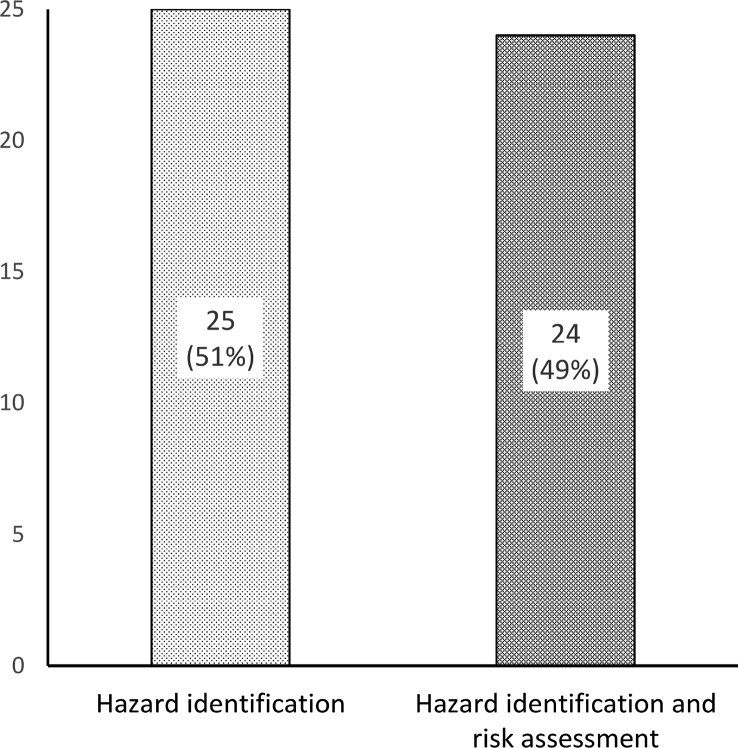
The aim of JSA. JSA, job safety analysis.

JSA has been used to fulfill a wide range of objectives. As depicted in Fig. 5, 25 articles (51%) used JSA for hazard identification and 24 articles (49%) used it for both hazard identification and risk assessment. Assigning appropriate control measures and hazard/risk communication is the ultimate and general objective of JSA, and most studies have also been conducted to achieve this goal (38 articles, 78%). However, there are a number of studies conducted JSA to accomplish specific objectives, including the selection appropriate personal protective equipment (PPE), developing a special safety program, assessing the effectiveness of interventional programs, and so on. Details are presented in Table 2.

**Table 2:** A summary of objectives of articles reviewed in this study

Articles	Objective	Number (%)
[9,13,26,27,[29], [30], [31], [32],[34], [35], [36], [37],^14^,[41], [42], [43], [44], [45],[47], [48], [49], [50], [51],^16^,[53], [54], [55], [56],^59^,^60^,^17^,^18^,[21], [22], [23],^25^]	Hazard identification/risk assessment/assigning appropriate control measures	38 (78%)
[38,52]	Assessing effectiveness of interventions	2 (4%)
[39,46]	Selection of appropriate PPE∗	2 (4%)
[20]	Incident investigation	1 (2%)
[40]	Developing respiratory protection program	1 (2%)
[57]	Determining emergency planning needs	1 (2%)
[33]	Total performance improvement	1 (2%)
[24]	Managing permit-to-work system	1 (2%)
[19]	Developing safety procedures	1 (2%)
[15]	Improving resilience	1 (2%)

PPE, personal protective equipment.

### 3.3 Risk assessment

**Fig. 6:**
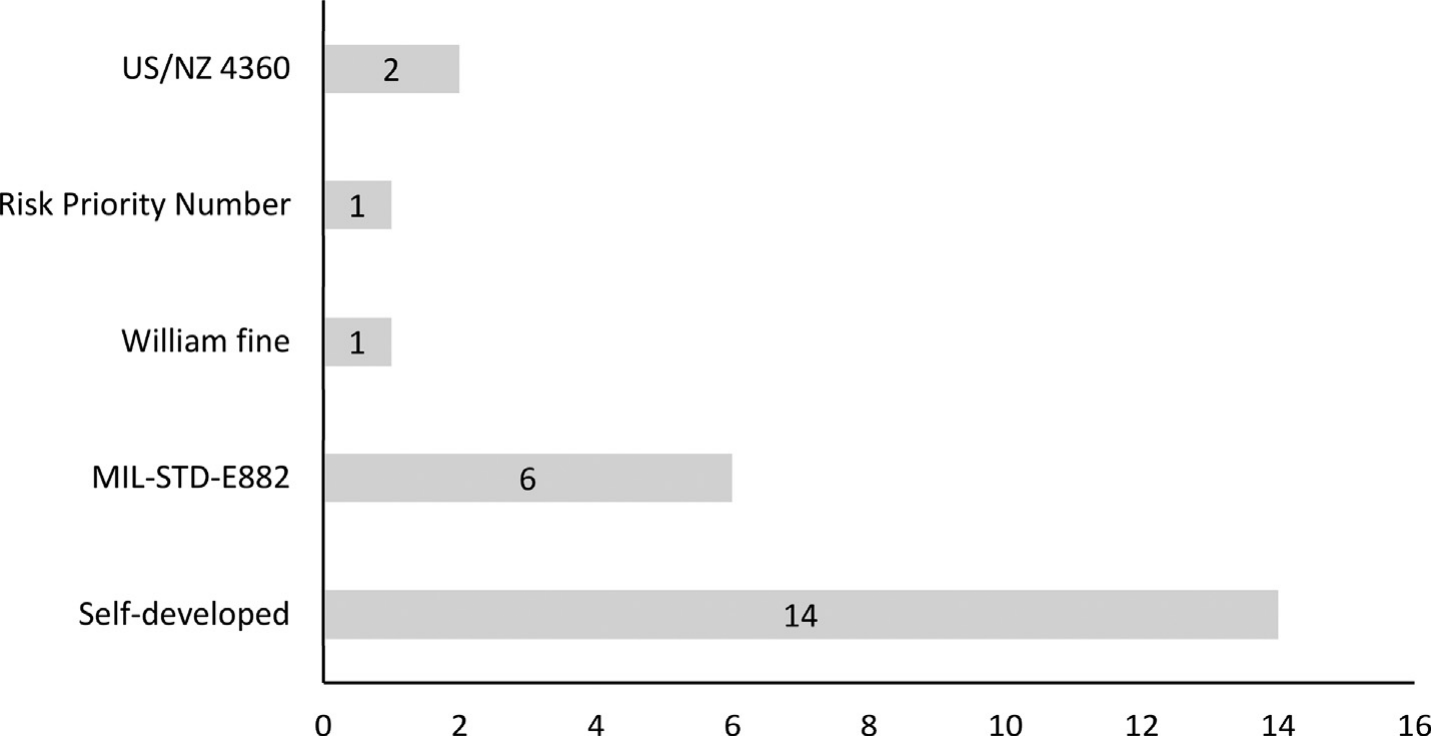
Methods used by articles for the risk assessment of hazards identified during JSA. JSA, job safety analysis.

Twenty-four articles performed risk assessment based on JSA results. A wide range of risk assessment methods has been employed by these studies (details are presented in Table 1). According to Fig. 6, 14 articles used self-developed methods, six articles adopted the MIL-STD-E882 standard, two studies used US/NZ 4360 standard, one study used Risk Priority Number (RPN) from Failure Modes and Effects Analysis method, and one study used the William Fine approach.

### 3.4 Shortcomings of JSA

This review reveals that JSA suffers from a number of important shortcomings and limitations. In this study, these shortcomings were categorized into two main groups: (1) those directly mentioned in the studies and (2) those that were inferred by reviewing all articles. A summary is available in Table 3. As evident, being tedious and time-consuming to perform was the most frequent drawback mentioned by studies. Lack of a comprehensive initial list of hazards is another important shortcoming which can create many problems in practice. Other shortcomings mentioned by studies are presented in Table 3.

**Table 3:** A summary of shortcomings found in or reported by previous studies

Article(s)	Shortcoming/limitation
[22,30,31,[34], [35], [36]]	Carrying out JSA sometimes is time-consuming and tedious, particularly at construction sites where operations, equipment, and working environment are continuously changing.
[15]	JSA does not consider recovery measures needed to bring the system back into the normal state.
[17]	JSA does not identify hazards emerged from performing operations out of the predefined time sequence.
[19]	JSA does not account for the cumulative characteristics of risks.
[29]	Lack of an initial comprehensive list of hazards in the traditional JSA can result in missing many notable hazards.
Inferred	JSA does not offer a unified framework for risk assessment and, as a result, studies have used a diversity of techniques for this purpose.
Inferred	Employees' participation is necessary when JSA is carried out. This important principle is sometimes ignored by practitioners. Employees' participation can ensure the throughout hazard identification.
Inferred	There are many instances in which the health and safety of workers are adversely affected by nearby activities performed by other workers. Such hazards should be considered in JSA.
Inferred	JSA does not emphasized on the hierarchy of controls in selecting control measures. Practitioners should always consider this hierarchy in recommending and selecting control measures.

JSA, Job Safety Analysis.

Among the shortcomings inferred by reviewing all articles is the lack of a universally accepted risk assessment framework. Consequently, as demonstrated in Table 1, a diversity of risk assessment methods are observed in the literature. In addition, the participation of front-line employees can help the JSA team to discover hazards more comprehensively. However, many studies did not mention whether they asked employees' opinions during JSA or not. Out of 49 included articles, only 18 cases (37%) mentioned that JSA had been conducted by a team. Another weak point of JSA is its inability to assess the effect of hazards posed by nearby activities. In fact, JSA is carried out based on the general working environment and ignores nonroutine/routine conditions in which the health and safety of workers are threatened by surrounding activities.

### 3.5 Advances in JSA

Reviewing articles published in this field demonstrates interesting advances in JSA during recent years. New advances are made to solve shortcomings of the traditional JSA and to add new properties to it. A summary of advances made by studies is presented in Table 4. Many researchers have made attempts to promote JSA by integrating it with other techniques and approaches, such building information system (BIM), Bayesian network, ontology, Petri net, resilience engineering principles, and so on. As a response to being time-consuming, artificial intelligence has been extensively used to perform JSA in an automatic or semi-automatic manner. Ontology principles have been used in conjunction with artificial intelligence tools to discern tasks and hazards in a more effective manner.

**Table 4:** A summary of recent advances in JSA

Article	Advances in JSA
[25]	Adding JSA plug-in to the building information modeling (BIM) software.
[15]	Integrating JSA and resilience engineering to develop a proactive risk assessment method. The new version includes recovery measures, known as “resilient-oriented” measures developed based on the resilience engineering principles, needed to bring the system back into the normal state.
[14]	Integrating JSA and Bayesian network for developing a new method for risk assessment of maintenance activities of subsea pipelines.
[17]	Integrating JSA and Petri net to develop a risk assessment method for non-routine operations in industries and to consider hazards arisen from performing such operations out of the predefined time sequence.
[29]	Developing a new version of JSA known as energy based JSA (EBJSA). The new approach was able to identify hazards more comprehensively.
[19]	Developing “job hazard dynamic assessment” to take into account the concept of risk propagation across a sequence of task steps. The new version takes into account the cumulative characteristics of risk across a sequence of tasks.
[30]	Integrating ontology, BIM, and JSA to develop a tool for automatic identification of hazards in construction projects.
[34]	Employing document modeling and ontology to facilitate conducting JSA.
[31]	Using ontology-based text classification to improve JSA.
[36]	Developing a new version of JSA known as CJSA to be used specifically at construction sites.
[35]	Upgrading JSA using the computer image generation for job simulation (CIGJS) method. The new version is able to model all types of interactions between human and working environment.
[22]	Proposing a new version of JSA which is less time-consuming. A “checklist of assignments” are developed to make the hazard identification process easier.
[33]	Integrating JSA and lean Kaizen to improve overall performance at homebuilding industries.

## 4 Discussion

In this study, we systematically reviewed original research articles conducted on JSA. The results demonstrate that JSA has found applications in many domains. Construction, process industries, and healthcare are the main sectors in which JSA has been implemented. This review demonstrates that JSA suffers from a number of serious shortcomings. Being time-consuming has been mentioned by many studies as a drawback of the method, making supervisors and employees reluctant to conduct the method and use its benefits before starting the work. This issue is more prominent at construction sites where frequent JSAs are needed because of changing nature of working conditions and equipment. Artificial intelligence has been introduced as a solution. Zhang et al [30] integrated ontology, BIM, and JSA to develop a tool for automatic hazard identification and safety planning at construction sites. Ontology is a way of knowledge representation in a particular domain by grouping relevant objects (or concepts) and making semantic relationships among them [61,62]. Three ontology models were used to develop this tool, each of which contained a distinct group of construction information. The output of the tool is a JSA and 4D model of the construction site demonstrating which type of safety measures are required at each phase of the project. Wang and Boukamp [34] and Chi et al [31] were other studies attempting to improve JSA using ontology. Wang and Boukamp [34] made attempts to make JSA less time-consuming by integrating document modeling and ontology principles. The aim was to produce new JSA documents based on a collection of available documents or a knowledge base according to their semantic properties. JSA documents from a private construction company were used to test the model. Authors claimed that the proposed framework can quickly modify the existing JSA documents as working condition changes. Chi et al [31] explained that in order to assign effective control measures, each task scenario must be investigated using JSA separately because of differences in working situations. For example, working at height can take many forms which need totally different control measures. The authors proposed a “semi-automated” JSA based on ontology-based text classification (TC). The CPWR database and the NIOSH FACE program are used for testing and training the model, respectively. The authors stated that deficiencies in training documents resulted in unsatisfactory model performance.

Rozenfeld et al [36] developed a new version of JSA known as construction JSA (CJSA). The Construction Hazard Assessment with Spatial and Temporal Exposure (CHASTE) approach [63] plays a central role in CJSA. CHASTE is a knowledge base of construction activities and related “loss-of-control events,” designed based on the assumption that core activities and tasks are similar at all construction sites, but the risk level is different as it is a function of work location, exposure to other teams, work method, and personal factors. CJSA has three main steps: first, all activities at the construction site and relevant loss-of-control events are identified. Second, the occurrence likelihood of each loss-of-control event, possible intensifying factors, and probability of using personal protective equipment. Third, the magnitude of consequences is estimated for loss-of-control events. Patrucco et al [35] employed the computer image generation for job simulation (CIGJS) method to promote the effectiveness and usability of the conventional JSA. A 3D interactive environment built by CIGJS covered all types of interactions between humans and the surrounding environment by considering anthropometric characteristics of humans, physical dimensions of machinery and buildings, physical laws, weights, frictions, forces, and so on. So, all actions of an employee can be simulated and the possibility of direct contact with dangerous parts of machinery can be detected. Combining JSA and CIGJS is very effective in visualizing hazards threatening the health and safety of a worker performing a particular task. In the same vein, Veley et al [22] also argued traditional JSA for being tedious and time-consuming, which can negatively affects its usefulness. To make JSA less time-consuming, Veley et al [22] recommended a JSA-based “checklist of assignments” to be completed before starting the task/work. Past experiences and other useful sources are used to identify possible hazards created by each step of the work. Assignment includes installing, checking, inspecting, and testing the required control measures and supervision required to perform the work safely. Authors claimed that this approach tremendously reduces the time needed to conduct JSA.

However, using artificial intelligence in safety assessment should be with caution because in some cases, it can be misleading and unable to identify all hazards. Although artificial intelligence supersedes humans in many tasks, in some cases, it needs big data or some levels of simplicity to work correctly [64]. In other words, there is no guarantee that these automated safety assessment methods would be able to find out all hazards posed by a task or recommend the most effective control measures. In this regard, Chi et al [31], who developed a semi-automated JSA based on ontology, reported that deficiencies in training data reduced the model performance significantly. Moreover, JSA is normally performed by a team composed of safety experts and employees, using artificial intelligence for automating JSA would remove employees' participation.

Another important shortcoming of JSA was the lack of a comprehensive list of hazards, which can result in missing some important hazards. In this regard, Zheng et al [29] proposed the energy-based JSA (EBJSA) for covering this drawback. The method is inspired by the “energy release theory” and is based on the promise that unwanted release of energy can cause damage to the surrounding environment, equipment, and people [65]. According to EBJSA, all sources of energy (gravity, motion, mechanical, electrical, pressure, temperature, chemical, biological, radiation, and sound) and arising hazards in each step of a task must be discerned. Next, 10 different strategies, according to the “energy transfer model,” can be adopted to prevent the incident or mitigate its consequences [66]. However, it still does not cover all hazards. For example, exposure to workplace violence may be missed when JSA is performed for nurses. Therefore, it seems necessary to provide a context-based comprehensive list of hazards before starting JSA. Brainstorming sessions, reviewing available documents, and previous incidents are useful for developing such a list. Moreover, JSA should be conducted with a team composed of safety experts, supervisors, and employees. In other words, the participation of employees performing the task is a necessity to ensure a more comprehensive identification of hazards.

Previous studies have not used a consistent risk assessment method. Inconsistency in risk assessment methods is a major weak point of JSA. As a consequence of such inconsistency, different construction sites cannot compare their results with each other and they cannot use their experiences regarding control measures because it is not clear how much these control measures are effective in reducing risk levels. In addition, there is a possibility to use a nonstandard method for risk assessment. There are several standard and well-accepted risk matrix that can be used in this respect. The risk matrix recommended by the hazards and effects management process (HEMP) has been regarded by a number of studies as an effective and easy-to-use tool [67,68].

Another important shortcoming of JSA is the ignorance of hazards posed by surrounding activities. There are many instances in which the health and safety of employees are threatened by activities carried out in the vicinity of the employee. As JSA focuses on the task performed by the employee, such hazards may be missed even though they can have an adverse impact on the health and safety of the employee. Therefore, it is important to consider hazards posed by such activities when conducting JSA.

The ultimate goal of JSA is to control hazards. There may be a variety of options for controlling identified hazards that are different in effectiveness, reliability, and feasibility. Selecting the best option may be confusing if the hierarchy of controls is not taken into account. The hierarchy provides practitioners and health and safety experts with a guide to select the most appropriate control measures. In this study, the importance of using this hierarchy was only mentioned by three studies [29,35,48]. The hierarchy recommends selecting control measures in the following order of priority: (1) eliminating the hazard, (2) substituting the hazard with a less intense one, (3) implementing engineering control measures, (4) adopting administrative control measures, and (5) using PPE.

## 5 Conclusion

JSA is increasingly used in various domains for hazard identification and risk assessment. Being time consuming and tedious to perform is the most important factor impeding its use in industries. Artificial intelligence is the promising tool to solve this problem. A comprehensive JSA covering all these weak points should have at least seven steps: (1) selecting a task, (2) developing a comprehensive list of hazards based on brainstorming sessions, reviewing available documents, and previous incidents, (3) analyzing the task and decomposing it in subtasks and steps, (4) determining nearby routine/non-routine activities, (5) identifying all hazards posed by subtask itself and surrounding routine and non-routine activities, considering the time sequence tasks (6) risk assessment using a unified method, and (7) selecting control measures preferably based on the hierarchy of controls.

## Conflicts of interest

There is no conflict of interest to declare.
